# Prognostic and immune infiltration features of disulfidptosis-related subtypes in breast cancer

**DOI:** 10.1186/s12905-023-02823-0

**Published:** 2024-01-02

**Authors:** Sheng Chen, Xiangrong Li, Wen Ao

**Affiliations:** Oncology Department III, The Central Hospital of Xiaogan, No.6, Guangchang Road, Xiaogan City, 432000 Hubei Province China

**Keywords:** Disulfidptosis, Breast cancer, Unsupervised clustering, Immune feature, Immunotherapy

## Abstract

Breast cancer (BC) is a prominent cause of cancer incidence and mortality around the world. Disulfidptosis, a type of cell death, can induce tumor cell death. The purpose of this study was to analyze the potential impact of disulfidptosis-related genes (DRGs) on the prognosis and immune infiltration features of BC. Based on DRGs, we conducted an unsupervised clustering analysis on gene expression data of BC in TCGA-BRCA dataset and identified two BC subtypes, cluster1 and cluster2, with cluster1 showing a higher likelihood of favorable survival. Through immune analysis, we found that cluster1 had lower proportions of infiltration in immune-related cells, including aDCs, DCs, NK_cells, Th2_cells, and Treg. Based on the immunophenoscore (IPS) results, we inferred that cluster1 might benefit more from immune checkpoint inhibitors targeting CTLA-4 and PD1. Targeted small molecule prediction results showed that patients with cluster2 BC might respond better to antagonistic small molecule compounds, including clofazimine, lenalidomide, and epigallocatechin. Differentially expressed genes between the two subtypes were found to be enriched in signaling pathways related to steroid hormone biosynthesis, ovarian steroidogenesis, and neutrophil extracellular trap formation, according to enrichment analyses. In conclusion, this study identified BC subtypes based on DRGs so as to help predict patient prognosis and provide valuable tools for guiding clinical management and precise treatment of BC patients.

**Supplementary Information:**

The online version contains supplementary material available at 10.1186/s12905-023-02823-0.

## Introduction

Breast cancer (BC) has exceeded lung cancer to become the most common cancer in women worldwide [1]. Male BC is becoming more common worldwide, despite its rarity [2]. Genetics is the most important risk factor for BC [3]. Despite various treatments including immunotherapy, targeted therapy, chemotherapy, radiation therapy, and surgery, BC patients are prone to developing drug resistance [3]. Since BC is a heterogeneous disease with different molecular subtypes and biological characteristics, treatment should be tailored to each patient based on molecular features [4]. Therefore, innovating reliable and effective prognostic biomarkers is urgently needed in order to identify different subgroups of BC patients and instruct precise and personalized treatment.

One of the significant hallmarks of cancer is Metabolic reprogramming. Recently, Liu et al. [5] revealed disulfidptosis, a metabolism-related form of regulated cell death (RCD). Differing from conventional forms of programmed cell death such as apoptosis, ferroptosis, and necroptosis, disulfidptosis is a novel form of programmed cell death triggered by disulfide stress. It is featured by the collapse of cellular cytoskeleton proteins and F-actin due to the intracellular accumulation of disulfide bonds [6]. Liu et al. [5] also identified four genes as promoters of disulfidptosis: solute carrier family 7 member 11 (SLC7A11), solute carrier family 3 member 2 (SLC3A2), recombinant ribophorin 1 (RPN1), and NCK-associated protein 1 (NCKAP1),. Among these four genes, SLC7A11 has received a lot of attention. When glucose is limited, tumor cells overexpressing SLC7A11 rapidly deplete NADPH and accumulate disulfides abnormally, inducing disulfidptosis [6]. Inducing cell death is well-known to be a potential therapeutic approach for cancer treatment. Understanding the mechanisms of cell death and their application in the treatment of cancer patients is therefore of great significance. Several studies have demonstrated the significant role of disulfidptosis in the prognosis and immune response of various cancers, including bladder cancer [7], renal cell carcinoma [8], lung adenocarcinoma [9], and cervical cancer [10]. However, limited research has been conducted on the impact mechanism of disulfidptosis on the prognostic survival and immune infiltration features of BC.

In this study, we intended to identify different subtypes of BC based on disulfidptosis-related genes (DRGs) and analyze the characteristics of disulfidptosis-related BC subtypes from the perspectives of tumor mutation, immune infiltration, immunotherapy response prediction, and targeted small molecule prediction. Through enrichment analyses, we analyzed the biological functions of differentially expressed genes (DEGs) related to disulfidptosis-related BC subtypes. Our findings provide valuable insights for future investigations of disulfidptosis in BC.

## Materials and methods

### Data download

We downloaded the expression data and clinical data of BC (TCGA-BRCA) from The Cancer Genome Atlas (TCGA) database (https://portal.gdc.cancer.gov/). 1,113 BC tumor samples were obtained, and after excluding patient samples with a survival time of less than 30 days or those with incomplete clinical information, 1,044 BC samples remained. In addition, based on previous studie [5], we identified four DRGs, namely SLC7A11, SLC3A2, RPN1, and NCKAP1.

### Unsupervised clustering

The target genes were subjected to unsupervised clustering analysis using the hierarchical consensus clustering algorithm in the ConsensusClusterPlus package. The analysis was repeated 1,000 times to ensure the stability of the classification, determine the optimal number of clusters, and identify different clusters.

### Tumor mutation analysis

The SNV mutation data of BC were detected by using the maftools package to plot the mutation of different subtypes including mutation types, SNV class, and differences in mutation rate.

### Immune infiltration analysis

By using single sample gene set enrichment analysis (ssGSEA), we scored 28 types of immune cells in the tumor microenvironment (TME) of different subtypes to analyze the level of immune cell infiltration and immune function.

### Prediction of immunotherapy response

We downloaded the immunophenoscore (IPS) of BC patients from The Cancer Immunome Atlas (TCIA) (https://tcia.at) to predict the response of BC patients to immune checkpoint inhibitor (ICI) therapy. A higher IPS result indicated that BC patients responded better to ICI therapy.

### Differential analysis

We applied edgeR package to perform differential expression analysis between different subtypes and selected DEGs between subtypes according to the threshold (FDR < 0.05, logFC > 1).

### Targeted small molecule prediction

The cMAP (https://clue.io/) is a biological application database with disruptors, gene expression, and diseases interconnected. It can be used to identify drugs with high correlation to the disease by comparing them to gene expression profiles, infer the main structure of most drug molecules, and induce the possible mechanisms of drug molecules. The top 150 DEGs between subgroups were input into the cMAP database to predict targeted small molecule compounds with better therapeutic effects.

### Protein-protein interaction (PPI) network

We used STRING database (https://cn.string-db.org/cgi/input.pl) to construct a PPI network of DEGs. Interactions with confidence scores higher than 0.9 were selected.

### Gene ontology (GO) and kyoto encyclopedia of genes and genomes (KEGG) enrichment analyses

We conducted GO and KEGG [11–13] enrichment analyses (*p*-value < 0.05) on DEGs in subtypes by using clusterProfiler package. The biological functions and signaling pathways enriched by DEGs were analyzed.

### Tumor activity analysis

Genes were divided into high and low expression groups based on the median expression values. The ssGSEA algorithm was applied to calculate the Angiogenic activity score and Mesenchymal-epithelial-mesenchymal transition (EMT) score for each tumor sample, with the corresponding marker genes listed in Supplementary Table 1. Wilcoxon test was performed to compare the high and low expression groups, and visualization was conducted using the R package ggplot [14].

## Results

### Identification of DRG clusters in BC

**Fig. 1:**
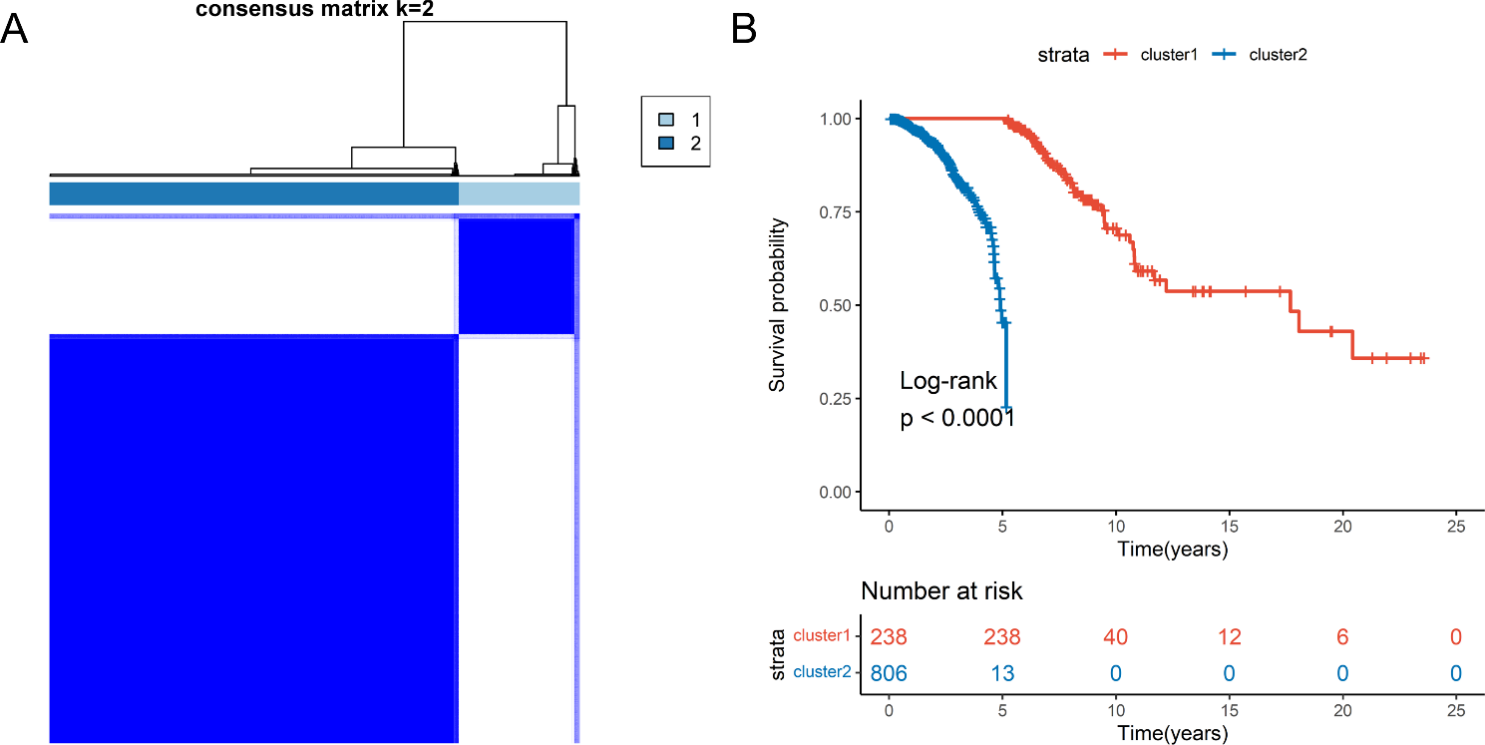
Identification of DRG clusters in BC based on DRGs **(A)** Clustering results of DRGs expression data in BC. The clustering result of patient samples when K = 2. **(B)** Survival analysis of different subtypes. *P* < 0.0001 indicates a significant difference in survival rates between patients in cluster1 and cluster2

To investigate the role of four disulfidptosis-related genes (SLC7A11, SLC3A2, RPN1, and NCKAP1) in cancer progression, we divided TCGA patients into high and low expression groups based on the median expression levels of these four genes. We investigated the Angiogenic activity score and Mesenchymal-EMT score in patients from different groups. The results revealed significant differences in both Angiogenic activity score and Mesenchymal-EMT score between the high and low expression groups (*P* < 0.05) (Figure S1), suggesting that disulfidptosis may play an important role in the progression of cancer. To comprehensively understand the expression patterns of disulfidptosis in BC, we performed an unsupervised clustering analysis on 1,044 BC samples based on four DRGs. According to the results of unsupervised clustering analysis, we divided the 1,044 BC samples into two clusters, cluster1 and cluster2, which contained 238 and 806 samples, respectively (Fig. 1A). Survival analysis revealed that the prognosis of cluster1 was greatly better than that of cluster2 (P < 0.0001) (Fig. 1B).

### Tumor mutation analysis

**Fig. 2:**
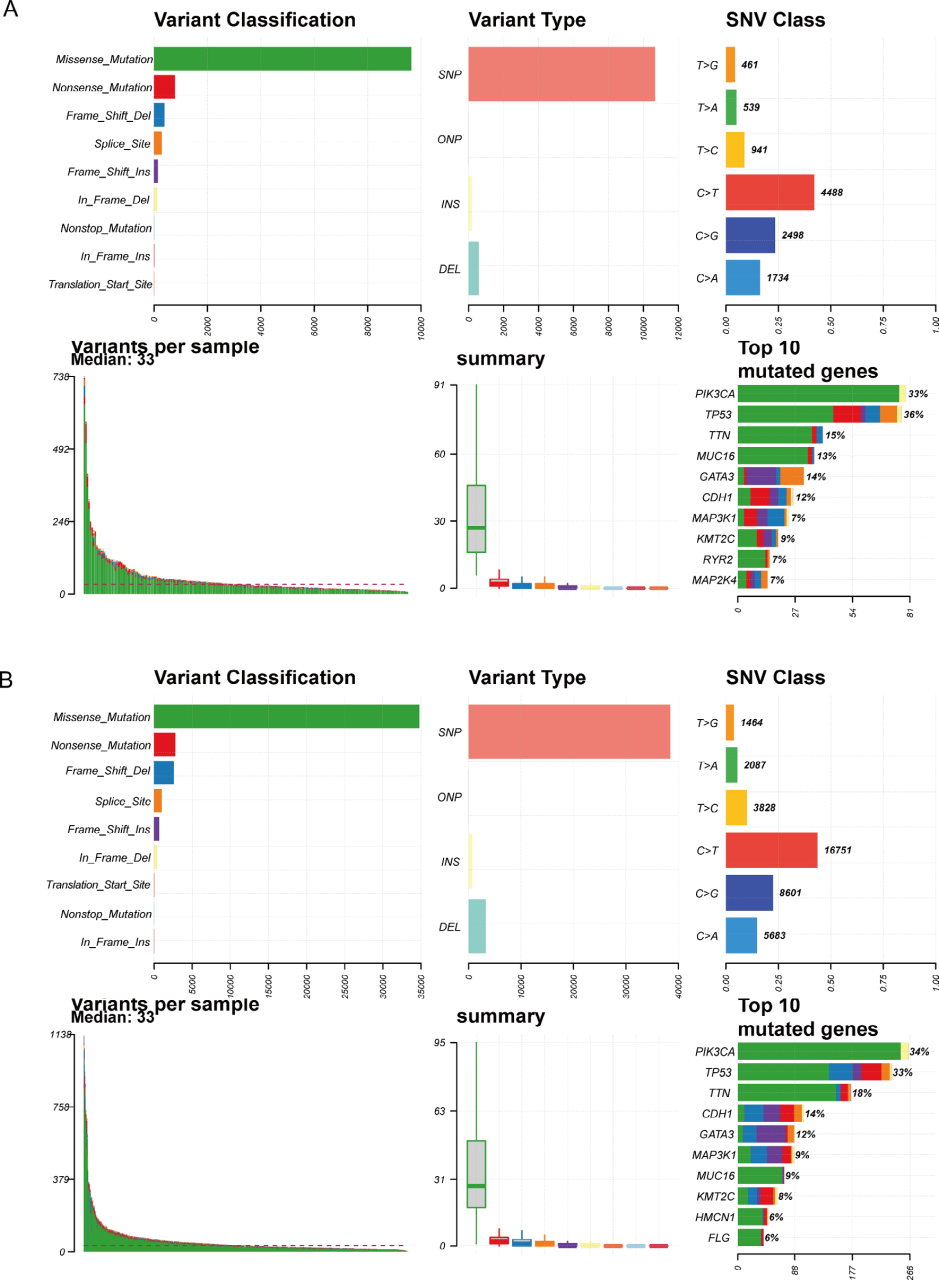
Gene mutation analysis of two BC subtypes **(A)** Mutation information of Cluster1. **(B)** Mutation information of Cluster2. In the variant classification plot, each mutation type is represented by a specific color. Variant type and SNV class represent the number of genomic variant types and base mutation types, respectively. Variants per sample indicates the types of mutations and their corresponding counts in each sample. The colors in the variant classification summary plot and the top 10 mutated genes plot correspond to the colors in the variant classification plot

Through analysis of tumor gene mutations, we observed that the two BC subtypes were mainly characterized by missense mutations, with SNP being the main mutation type. The top ten mutant genes in cluster1 were PIK3CA, TP53, TTN, MUC16, GATA3, CDH1, MAP3K1, KMT2C, RYR2, and MAP2K4, while the top 10 mutant genes in cluster2 were PIK3CA, TP53, TTN, CDH1, GATA3, MAP3K1, MUC16, KMT2C, HMCN1, and FLG (Fig. 2A-B).

### Immune infiltration analysis

**Fig. 3:**
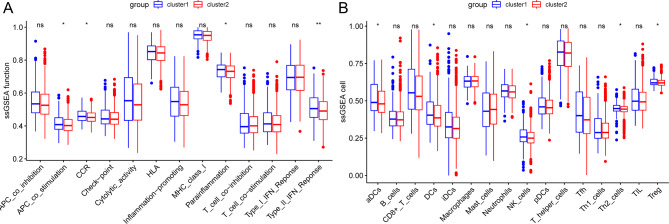
Immune analysis of the two subtypes of BC samples **(A)** Immune function analysis. **(B)** Immune cell infiltration analysis * *P* < 0.05; ns: no significance

To further discuss the different immune statuses between the two subtypes of BC, we evaluated the immune function and immune cell infiltration levels of both subtypes. The immune-related functions such as APC-co-stimulation, CCR, parainflammation, and Type_II_IFN Reponse were significantly higher in cluster1 than in cluster2（P＜0.05） (Fig. 3A). The immune-related cell infiltration proportions of aDCs, DCs, NK_cells, Th2_cells, and Treg were significantly higher in cluster1 than in cluster2（P＜0.05） (Fig. 3B).

### Prediction of immunotherapy response

**Fig. 4:**
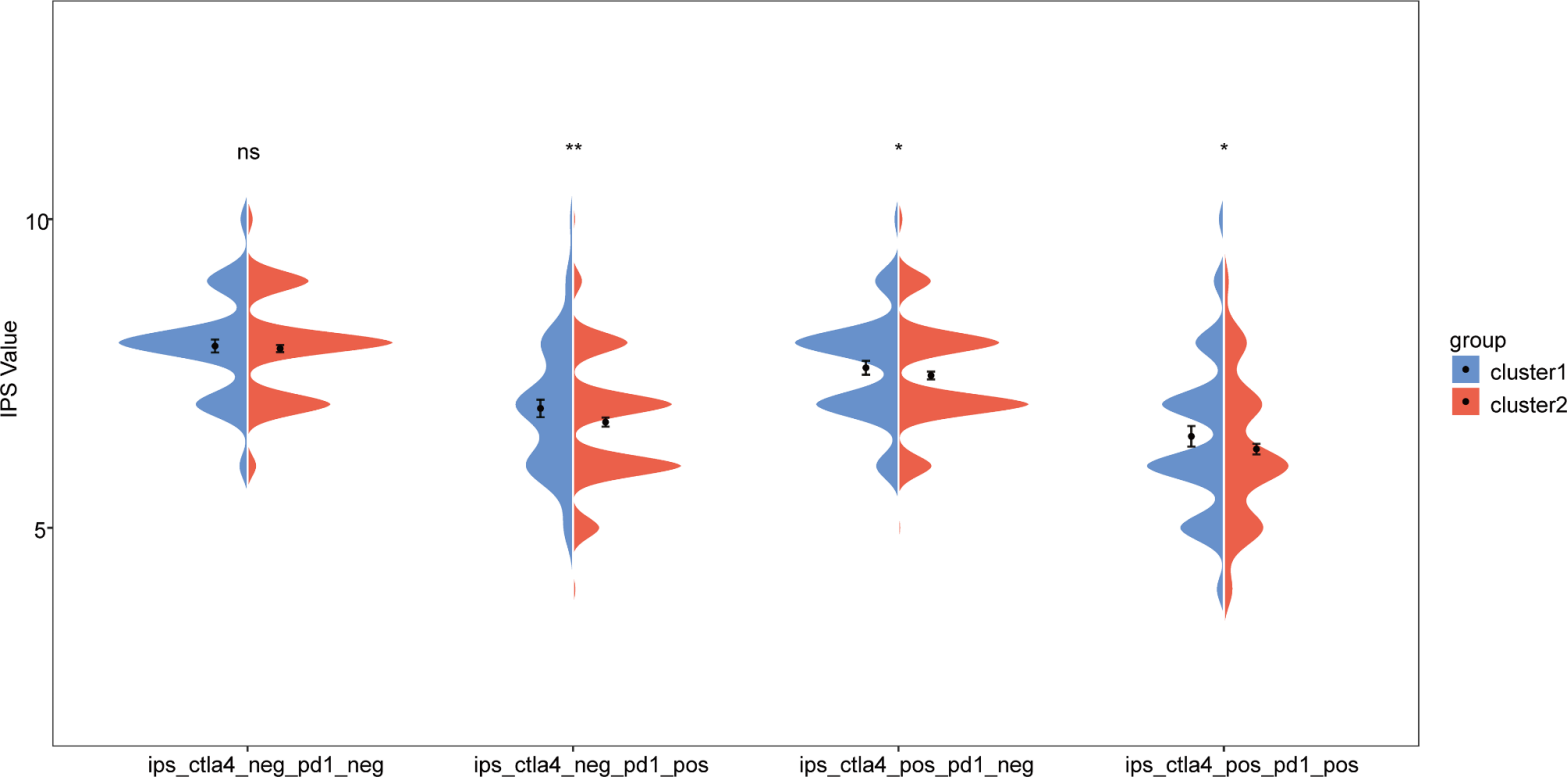
Violin plot of IPS results for the two BC subtypes PD1 and CTLA4 were enrolled for IPS analysis, including four parts: ips-ctla4-neg-pd1-neg (CTLA4 negative response and PD1 negative response); ips-ctla4-neg-pd1-pos (CTLA4 negative response and PD1 positive response); ips-ctla4-pos-pd1-neg (CTLA4 positive response and PD1 negative response); ips-ctla4-pos-pd1-pos (CTLA4 positive response and PD1 positive response); * *P* < 0.05; ** *P* < 0.01; ns: no significance

To detect the response of the two BC subtypes to immunotherapy, we analyzed the IPS scores of both subtypes. The IPS results showed that ips_ctla4_neg_pd1_pos, ips_ctla4_pos_pd1_neg, and ips_ctla4_pos_pd1_pos were significantly higher in cluster1 than in cluster2（P＜0.05） (Fig. 4), suggesting that BC patients in cluster1 were more likely to benefit from ICI therapy targeting cytotoxic T lymphocyte-associated antigen 4 (CTLA4) and programmed cell death protein 1 (PD-1).

### Prediction of BC targeted small molecules

We aimed to identify potential therapeutic drugs for cluster2 BC patients with poor prognosis. Firstly, we identified 585 DEGs for cluster1 and cluster2 (Supplementary Table 2), and predicted targeted small molecule compounds based on the top 150 upregulated DEGs in cluster2 (Table 1), which could be used as candidate drugs for treating cluster2 BC patients. From Table 1, clofazimine, lenalidomide, and epigallocatechin were the most likely antagonistic small molecule compounds predicted to have a response in cluster2 BC patients.

**Table 1:** Antagonistic small molecules in two subtypes of BC

Score	Type	ID	Name	Description
-99.93	cp.	BRD-K56614220	clofazimine	GK0582 inhibitor
-99.93	cp.	BRD-K05926469	lenalidomide	Antineoplastic
-99.93	cp.	BRD-K55591206	epigallocatechin	Nitric oxide synthase inhibitor

Note: “Score” represents the predictive score. “Type” refers to the classification in the database. “cp” denotes Compond database. “ID” represents the drug identifier. “Name” corresponds to the drug name. “Description” provides a description of the drug

### PPI network

**Fig. 5:**
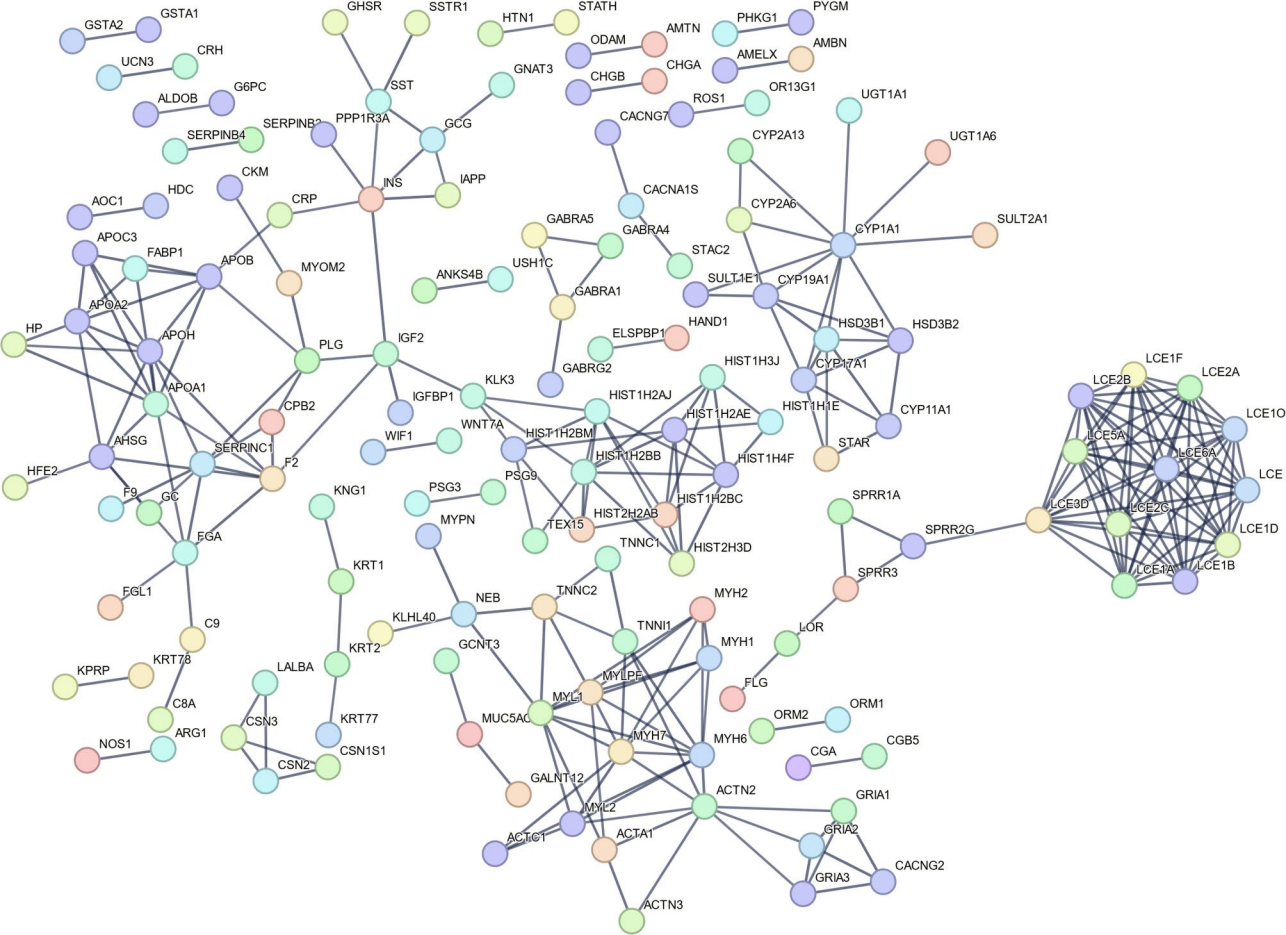
PPI network based on DEGs between the two subtypes of BC In the diagram, circles represent proteins, and the connecting lines represent interactions between proteins

Using the STRING database, we constructed a PPI network based on the DEGs between the two subtypes of BC (Fig. 5). The PPI network was made up of 151 nodes and 271 edges. The degree value of LCE1A was 11, while those of LCE1B, LCE1C, LCE1D, LCE1F, LCE2A, LCE2B, LCE2C, LCE2D, LCE3D, LCE5A, and LCE6A were 10.

### GO and KEGG enrichment analyses

**Fig. 6:**
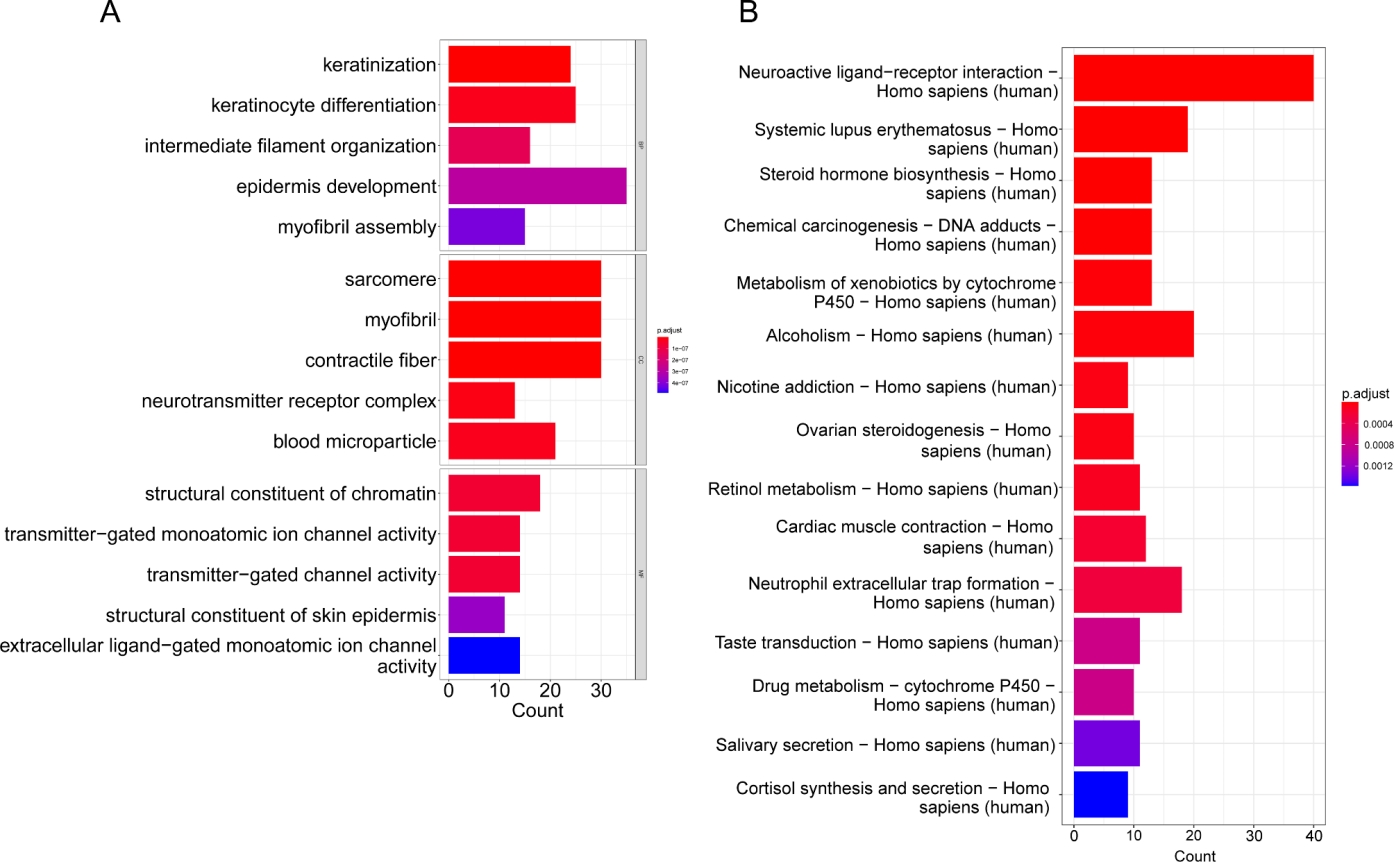
Enrichment analyses results of DEGs related to the two subtypes of BC associated with disulfidptosis **(A)** GO enrichment analysis of DEGs. **(B)** KEGG enrichment analysis of DEGs. The horizontal axis represents the number of genes enriched in the pathway, while the vertical axis represents the enriched function or pathway. The color gradient from red to blue indicates the decreasing significance of the enrichment, with red indicating strong enrichment and blue indicating weak enrichment

We performed GO and KEGG enrichment analyses on the DEGs between the two subtypes. GO analysis demonstrated that these genes were enriched in biological processes such as myofibril assembly, epidermis development, and cellular components such as contractile fiber, myofibril, and sarcomere, as well as molecular functions such as transmitter-gated channel activity, transmitter-gated monoatomic ion channel activity and structural constituent of chromatin (Fig. 6A). KEGG analysis displayed that these DEGs were enriched in signaling pathways such as neuroactive ligand-receptor interaction, systemic lupus erythematosus, steroid hormone biosynthesis, chemical carcinogenesis-DNA adducts, metabolism of xenobiotics by cytochrome P450, alcoholism, nicotine addiction, ovarian steroidogenesis, retinol metabolism, and neutrophil extracellular trap formation (Fig. 6B).

### Identification of DRG gene clusters in BC

**Fig. 7:**
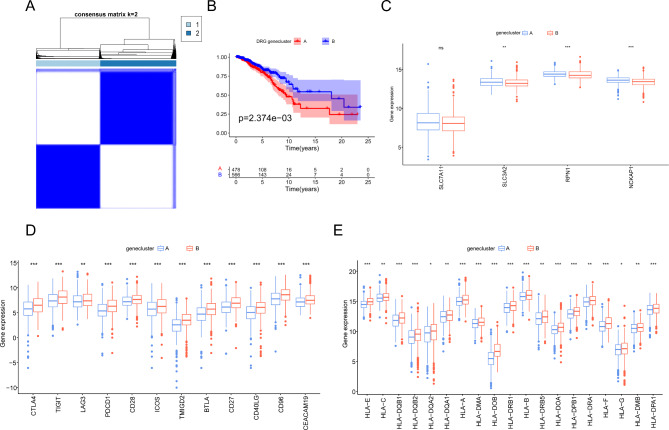
Identification of DRG gene clusters in BC **(A)** Unsupervised clustering based on DEGs between the two subtypes of BC associated with disulfidptosis. **(B)** Survival analysis of different gene clusters. **(C)** Expression analysis of SLC7A11, SLC3A2, RPN1, and NCKAP1 in different gene clusters. **(D)** Differential expression analysis of immune checkpoints in different gene clusters. **(E)** Differential expression analysis of HLA in different gene clusters. * *P* < 0.05; ** *P* < 0.01; *** *P* < 0.001; ns: no significance

To further investigate the potential biological behavior of the two subgroups related to disulfidptosis, we performed univariate Cox regression analysis on the DEGs between the two subgroups and identified 36 genes significantly related to prognosis (*P* < 0.05). Using these 36 genes, we performed unsupervised clustering analysis and identified two DRG gene clusters in BC patients (Fig. 7A). Patients in gene cluster A had a poorer prognosis than those in cluster B (Fig. 7B), indicating that they had worse clinical outcomes. The expression levels of SLC3A2, RPN1, and NCKAP1 were significantly higher in DRG gene cluster A than in gene cluster B（P＜0.05） (Fig. 7C), while the immune checkpoint and HLA were significantly lower in DRG gene cluster A than in gene cluster B (Fig. 7D-E).

## Discussion

Disulfide compounds are important for maintaining protein stability, and disulfidptosis is a type of cell death in tumor cells [6]. There are four genes related to disulfidptosis, including SLC7A11, SLC3A2, RPN1, and NCKAP1^15^. SLC7A11 and SLC3A2 are subunits of the cystine/glutamate antiporter xCT, and the expression of GPX4 driven by xCT determines the sensitivity of BC cells to ferroptosis inducers [16]. The anti-diabetic drug metformin induces ferroptosis in BC cells by suppressing the UFMylation of SLC7A11^17^. Obesity-associated protein (FTO) prevents thyroid cancer progression by SLC7A11 m6A methylation in a ferroptosis-dependent manner [18]. By targeting SLC7A11, miR-5096 can inhibit the development of BC [19]. RPN1 is a ribonucleoprotein, and upregulation of RPN1 expression in BC is related to poor prognosis [20]. NCKAP1, as a tumor inhibitor gene in hepatocellular carcinoma, can improve the prognosis of liver cancer patients by targeting Rb1/p53^21^. Ma et al. [22] reported that miR-140-5p inhibits the proliferation, migration, and invasion of vascular smooth muscle cells by targeting and suppressing the expression of NCKAP1. In this study, based on the classification of SLC7A11, SLC3A2, RPN1, and NCKAP1, the association between disulfidptosis and patient survival was demonstrated. Furthermore, differential gene classification was performed between two subtypes, and survival analysis and expression analysis further elucidated the correlation between Disulfidptosis-related genes and patient survival. Additionally, both the first and second clustering analyses indicated that patients with higher survival rates corresponded to higher immunogenicity (IPS score) or immune levels (expression of immune checkpoints). Overall, the second clustering analysis validated the results obtained from the first analysis effectively.

In this study, we observed that patients (cluster 1 and cluster B) with higher survival rates exhibited higher immune levels. This suggests a potential relationship between patient survival and immunity in BC. It is well-known that the infiltration of immune cells in the tumor microenvironment (TME) influences tumor development. Interestingly, our study found higher levels of immune-related cell infiltration, including aDCs, DCs, NK cells, Th2 cells, and Treg cells, in cluster1. Dendritic cells are typical antigen-presenting cells in the immune system that regulate T cell responses [23]. NK cells are the major effector cell type in innate immunity, and they can autonomously kill target cells during tumor initiation. However, long-term exposure to the TME leads to NK cells being in an immunosuppressive state, promoting tumor immune evasion and metastasis [24]. The higher proportion of NK cell infiltration in cluster2 with poorer prognosis may be one of the reasons for promoting tumor immune escape. Th2 cells are a type of helper T cell that can regulate tumor immunity, and targeting Th2 with montelukast, a drug that blocks Th2, effectively improves the response of BC patients to immune checkpoint blockade (ICB) therapy [25]. Treg cells enhance BC immune escape by integrating integrin αvβ8-mediated TGF-β activation [26], which is consistent with the results of this study showing a higher proportion of Treg cell infiltration in cluster2 with poorer prognosis. Therefore, differences in the infiltration proportions of dendritic cells, NK_cells, Th2_cells, and Treg cells may affect the prognosis of different BC subtypes.

In this study, we observed higher expression levels of immune checkpoints in cluster B, which was associated with higher survival rates. ICI therapy in immunotherapy blocks checkpoints to alleviate their inhibitory effects on immune cells, activate T cells, destroy cancer cells, and restore the body’s ability to resist tumors [27]. A clinical trial showed that pembrolizumab, an anti-PD-1 drug, effectively improved the efficacy of paclitaxel in BC patient treatment, but not all BC patients benefited from ICI therapy [28]. This study found that the likelihood of benefiting from ICI therapy was greater in cluster1 of BC subtypes, indicating that the disulfidptosis-related BC subtype was helpful in predicting the efficacy of ICI therapy in patients.

Our study indicated that clofazimine, lenalidomide, and epigallocatechin had good therapeutic effects on cluster2 BC patients. Clofazimine is a targeted inhibitor of the Wnt signaling pathway, which has clinical value for triple-negative breast cancer with over-activated Wnt signaling pathway [29, 30]. Lenalidomide is an immunomodulatory drug that has been validated in the treatment of lymphoma and multiple myeloma [31, 32]. Epigallocatechin is a derivative of green tea catechins, and current studies have pointed out that epigallocatechin-3-gallate has application value in the prevention and treatment of BC [33]. Therefore, clofazimine and epigallocatechin have application values in the treatment of BC, while lenalidomide has not yet been validated for BC treatment.

The DEGs between the two BC subtypes are enriched in steroid hormone biosynthesis and ovarian steroidogenesis signaling pathways. Steroid hormones can regulate cellular, tissue, and organ functions throughout the human lifespan [34]. Postmenopausal women are more likely to develop BC when sex steroid hormones such as dehydroepiandrosterone sulfate, estradiol, and testosterone increase [35]. The ovarian steroid 17β-estradiol (E2) is an effective growth promoter for BC [36]. In addition, the DEGs between the two BC subtypes are related to the signaling pathway of neutrophil extracellular trap formation. Neutrophil extracellular traps take a pivotal part in BC invasion, evasion, and metastasis [37, 38]. We speculated that the different prognostic survival characteristics exhibited by the two BC subtypes might be related to steroid hormone biosynthesis, ovarian steroidogenesis, and neutrophil extracellular trap formation signaling pathways.

Although our study results were based on the analysis of DRGs to identify the potential subtypes of BC patients and reveal the characteristics of each subtype, changes in gene expression might not necessarily be genetically driven and they might be influenced by environmental factors. In addition, we obtained our results through bioinformatics analysis, and these results needed further experimental validations.

### Electronic supplementary material

Below is the link to the electronic supplementary material.

Supplementary Material 1

Supplementary Material 2

Supplementary Material 3

Supplementary Material 4

## Data Availability

The data that support the findings of this study are available from [https://portal.gdc.cancer.gov/, https://tcia.at, https://clue.io/, https://cn.string-db.org/cgi/input.pl].
