# Supplementary figures and images for: Prognostic and immune infiltration features of disulfidptosis-related subtypes in breast cancer
Source: BMC Womens Health. 2024 Jan 2;24:6. doi: 10.1186/s12905-023-02823-0 (PMC10763228; doi:10.1186/s12905-023-02823-0)

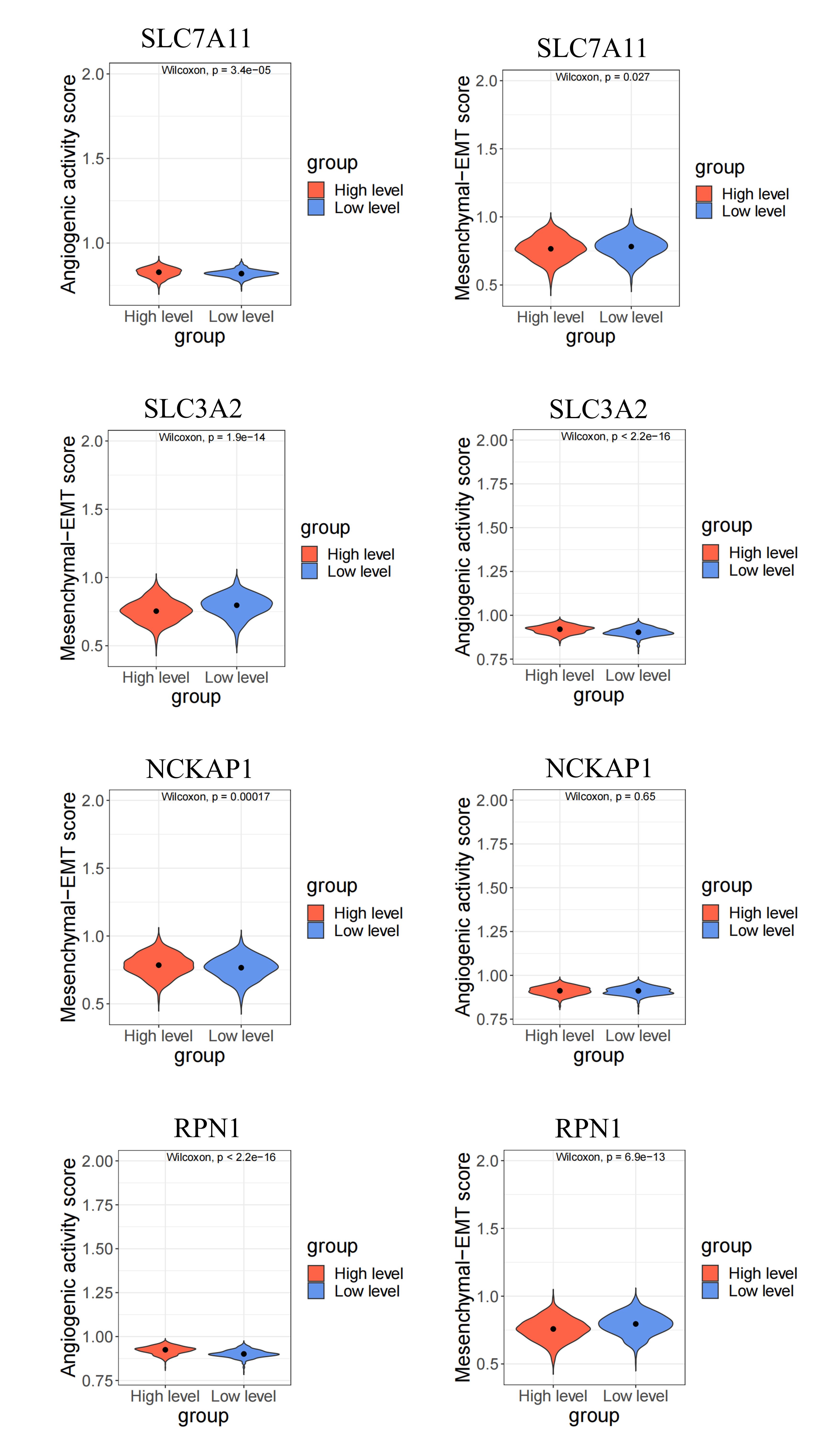

Supplement: Supplementary file 3 — Supplementary Material 3 [file 12905_2023_2823_MOESM3_ESM.tif]
