# Supplementary material for: Prognostic and immune infiltration features of disulfidptosis-related subtypes in breast cancer
Source: BMC Womens Health. 2024 Jan 2;24:6. doi: 10.1186/s12905-023-02823-0 (PMC10763228; doi:10.1186/s12905-023-02823-0)
Supplement: Supplementary file 4 — Supplementary Material 4 [file 12905_2023_2823_MOESM4_ESM.docx]

**Supplementary Figure 1** **The Angiogenic activity score and Mesenchymal-EMT score of the four disulfidptosis-related genes (SLC7A11, SLC3A2, RPN1, and NCKAP1) in the high and low expression groups**
